# Extinction vulnerability of tropical montane endemism from warming and upslope displacement: a preliminary appraisal for the highest massif in Madagascar

**DOI:** 10.1111/j.1365-2486.2008.01596.x

**Published:** 2008-08

**Authors:** CHRISTOPHER J RAXWORTHY, RICHARD G PEARSON, NIRHY RABIBISOA, ANDRY M RAKOTONDRAZAFY, JEAN-BAPTISTE RAMANAMANJATO, ACHILLE P RASELIMANANA, SHENGHAI WU, RONALD A NUSSBAUM, DÁITHÍ A STONE

**Affiliations:** * American Museum of Natural HistoryCentral Park West at 79th Street, New York, New York 10024-5192, USA; † Département de Biologie Animale, Université d'AntananarivoBP 906, Antananarivo (101), Madagascar; ‡ Department of Zoology, National Chung-Hsing University250 Kuo-Kwang Road, Taichung 402, Taiwan; § Museum of Zoology, University of MichiganAnn Arbor, Michigan 48109-1079, USA; ¶ Departments of Physics (AOPP) and Zoology, University of OxfordClarendon Laboratory, Parks Road, Oxford OX1 3PU, UK

**Keywords:** amphibia, conservation, distribution, elevation shifts, endemism, extinction, global warming, Madagascar, reptilia

## Abstract

One of the predicted biological responses to climate warming is the upslope displacement of species distributions. In the tropics, because montane assemblages frequently include local endemics that are distributed close to summits, these species may be especially vulnerable to experiencing complete habitat loss from warming. However, there is currently a dearth of information available for tropical regions. Here, we present a preliminary appraisal of this extinction threat using the herpetological assemblage of the Tsaratanana Massif in northern Madagascar (the island's highest massif), which is rich with montane endemism. We present meteorological evidence (individual and combined regional weather station data and reanalysis forecast data) for recent warming in Madagascar, and show that this trend is consistent with recent climate model simulations. Using standard moist adiabatic lapse rates, these observed meteorological warming trends in northern Madagascar predict upslope species displacement of 17–74 m per decade between 1993 and 2003. Over this same period, we also report preliminary data supporting a trend for upslope distribution movements, based on two surveys we completed at Tsaratanana. For 30 species, representing five families of reptiles and amphibians, we found overall mean shifts in elevational midpoint of 19–51 m upslope (mean lower elevation limit 29–114 m; mean upper elevation limit −8 to 53 m). We also found upslope trends in mean and median elevational observations in seven and six of nine species analysed. Phenological differences between these surveys do not appear to be substantial, but these upslope shifts are consistent with the predictions based on meteorological warming. An elevational range displacement analysis projects complete habitat loss for three species below the 2 °C ‘dangerous’ warming threshold. One of these species is not contracting its distribution, but the other two were not resampled in 2003. A preliminary review of the other massifs in Madagascar indicates potential similar vulnerability to habitat loss and upslope extinction. Consequently, we urgently recommend additional elevational surveys for these and other tropical montane assemblages, which should also include, when possible, the monitoring of local meteorological conditions and habitat change.

## Introduction

There is now substantial evidence for global warming and its biological consequences (Hughes, 2000; Walther *et al*., 2002; Parmesan & Yohe, 2003; Root *et al*., 2003; Karoly & Wu, 2005; Hegerl *et al*., 2007; Rosenzweig *et al*., 2007;). These biological responses include predictions of future species extinctions (Peterson *et al*., 2002; Williams *et al*., 2003; Thomas *et al*., 2004; Thuiller *et al*., 2005;) and empirical observations that link warming to extinctions (Pounds *et al*., 1999, 2006). However, to the best of our knowledge, globally only one field study has so far reported species extinctions that are associated with upslope distribution displacement, where species are pushed up and off the tops of mountains (Pounds *et al*., 1999). Upslope distribution shifts represent one of the biological fingerprints of global warming, when distributions move either in direct response to increasing temperature (Parmesan & Yohe, 2003; Root *et al*., 2003;), or in combination with other changes such as a lifting-cloud base (Pounds *et al*., 1999; Still *et al*., 1999;) or the availability of new habitat resulting from deglaciation (Seimon *et al*., 2007). Upslope displacement has been recently reported by multiple temperate studies (e.g. Grabherr *et al*., 1994; Parmesan, 1996; Pauli *et al*., 1996; Kullman, 2001; Erasmus *et al*., 2002; Epps *et al*., 2003; Konvicka *et al*., 2003; Pauli *et al*., 2007;), but there is currently very little information available for tropical regions (IPCC, 2007b).

Tropical montane regions typically exhibit high levels of local endemism, which may also include species confined to narrow elevational zones close to summits (Ricketts *et al*., 2005). Yet, to date, the vulnerability of most tropical montane assemblages to upslope extinction has not been well documented (Rull & Vegas-Vilarrúbia, 2006). Concerning tropical herpetological assemblages, a frog population census in Ecuador (1967–2003) found increases in the upper elevation limit in six of 76 surveyed species (Bustamante *et al*., 2005), and more generally for montane tropical frogs, a high incidence of ‘enigmatic’ declines (declines or disappearances in apparently intact primary habitats) has been suggested as a potential consequence of climate change or disease by the Global Amphibian Assessment (Stuart *et al*., 2004). More recently, Pounds *et al*. (2006) have reported evidence linking *Atelopus* frog extinctions to warming, and have suggested that a pathogenic chytrid fungus may be promoted by warming. Other studies have found strong evidence supporting emerging infectious diseases driving enigmatic amphibian declines in some tropical regions (e.g. Lips *et al*., 2005, 2006), or else gradual declines in herpetological assemblages that might possibly be linked to local climate change (Whitfield *et al*., 2007).

The biodiversity of Madagascar has long been recognized as one of the world's most threatened biotas, due to both the high levels of diversity and endemism on the island, and the decline of natural habitats (e.g. Jenkins, 1987; Myers *et al*., 2000; Ricketts *et al*., 2005;). The montane assemblages of Madagascar have been subject to periods of intense research (see Andriamialisoa & Langrand, 2003) and are remarkable for their high degree of regional endemism that is often specific to individual massifs. Although the species richness of assemblages is depressed at high elevations, a significant component of Madagascar's endemic species richness is confined to high montane environments (Jenkins, 1987). For example six of 35 *Calumma* chameleon species are endemic to zones within 600 m elevation of the highest summits (Raxworthy, in press; Raxworthy & Nussbaum, 2006;). Many of Madagascar's montane species might thus be potentially vulnerable to upslope distribution shifts from climate warming.

Although the evidence for global warming is well established (Hegerl *et al*., 2007; Trenberth *et al*., 2007;), regional warming trends in Madagascar have not been widely explored and the potential vulnerability of these endemic montane assemblages has not been discussed or studied. No observations of changes in physical or biological systems were noted for Madagascar in the latest Intergovernmental Panel on Climate Change report (Rosenzweig *et al*., 2007). The only specific studies for Madagascar known to us are Jury (2003) who reported an increase in coastal sea surface temperatures around Madagascar of ∼1 °C over the past century (see also Karoly & Wu, 2005) and Heiss *et al*. (1998) who detected a general warming trend (with oscillations) from 1930 to present in a core taken from a 350-year-old coral off the coast of southwest Madagascar. In addition, the widespread bleaching of coral reefs in Madagascar during 1988 has been linked to the exceptionally high (33 °C) ocean temperatures of this period (Wilkinson *et al*., 1999) and it has been predicted that the vegetation cover of Madagascar will show negative responses to a possible increasing frequency of El Niño Southern Oscillation (ENSO) phenomenon that is associated with global climate change (Ingram & Dawson, 2005).

**Fig. 1:**
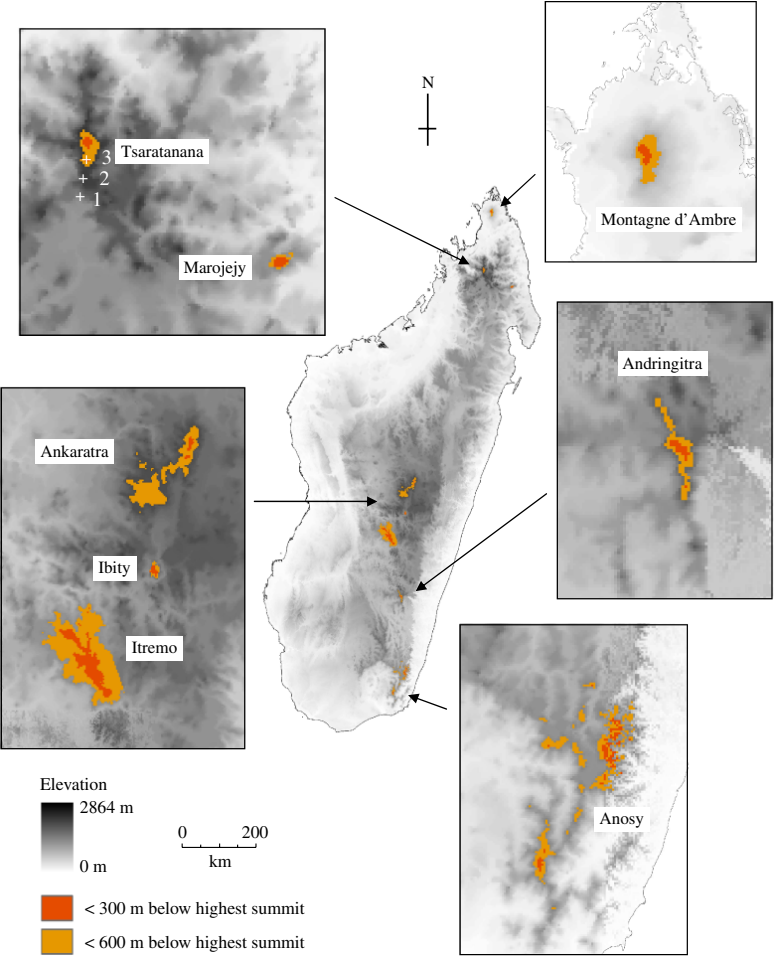
The distribution of montane habitats for the major massifs in Madagascar. Coloured areas represent habitats within 300 and 600 m elevation of the highest summits, for those massifs with known herpetological endemism. The three main camps on the Tsaratanana survey transect are numbered: (1) Befosa, (2) Matsabory Maiky, and (3) Bepia Ridge-Ambodinitsaratanana.

**Fig. 2:**
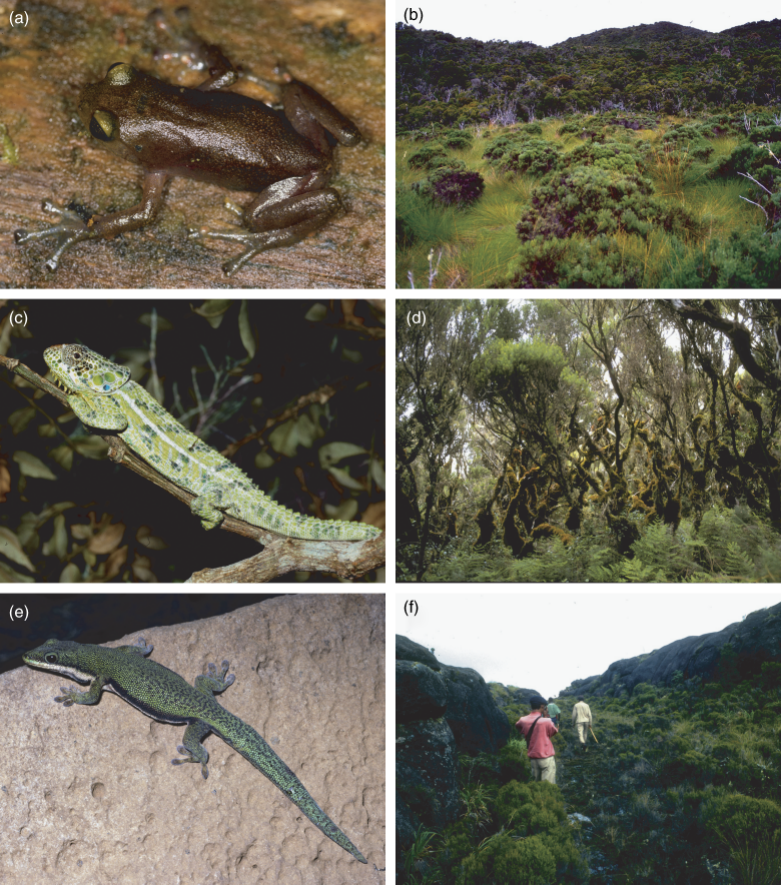
Example species and their associated habitats endemic to the summit region of Tsaratanana; (a) *Platypelis alticola*; (b) grass wetland and forest margin at 2500 m; (c) *Calumma tsaratananensis*; (d) forest at 2600 m; (e) *Phelsuma lineata punctulata*; (f) rocky outcrops and heathland at 2750 m.

**Fig. 3:**
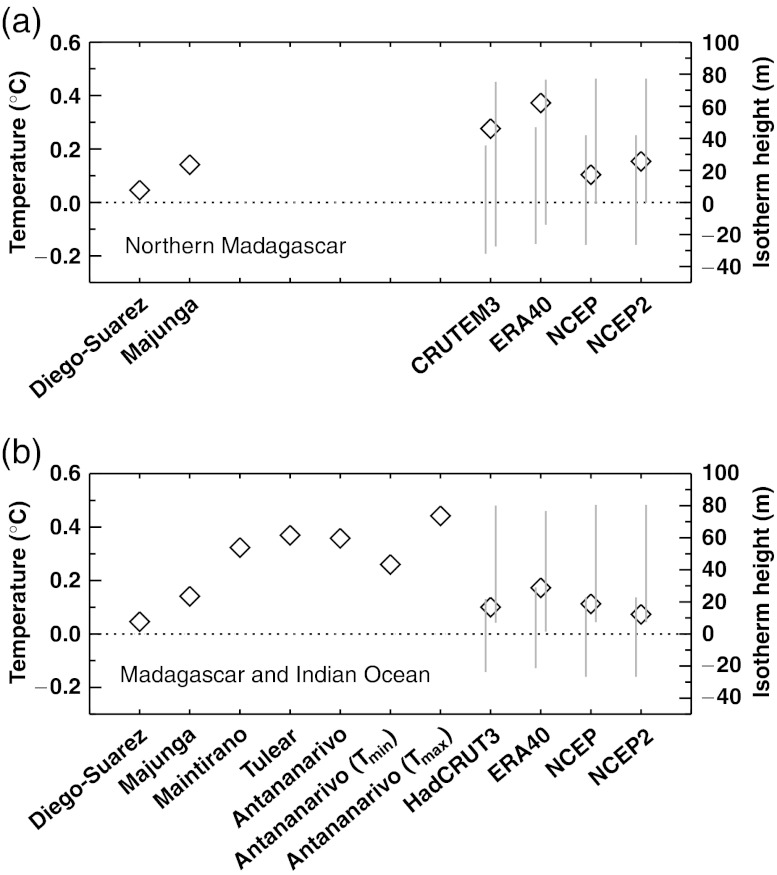
Changes in mean annual temperature between the decade 1984–1993 and 1994–2003 for five major towns in Madagascar and the gridded datasets. Right axis shows the corresponding change in the isotherm height assuming a lapse rate of 6°C per 1000 m. (a) Northern Madagascar: two northern towns (Diego-Suarez and Majunga) and the northern Madagascar regional land grid 46–51°E, 12–17°S; (b) Madagascar and the Malagasy Indian Ocean region: five towns distributed throughout Madagascar and the Madagascar regional land+ocean grid 42–54°E, 9–28°S. Values from observational datasets are plotted as diamonds. For each gridded observational dataset, values from model simulations (see ‘Materials and methods’) are plotted as lines denoting the approximate 5th–95th percentile range with control/historical simulations used for the left/right lines, respectively. Model data are retained for the calculation only where and when the respective observational dataset has data.

For this study, we selected the Tsaratanana Massif in northern Madagascar (sometimes referred to as the Northern Highlands) as the region of investigation (Fig. 1). We chose this massif for the following reasons: (1) the massif includes the highest summit in Madagascar (Maromokotro, 2876 m elevation, 14°01′S, 48°58′E); (2) the massif is rich in locally endemic species (e.g. Jenkins, 1987; Raxworthy & Nussbaum, 1997; Fig. 2); (3) the massif includes an intact transect of primary habitat between 1400 and 2876 m, and (4) during 1993 and 2003 we conducted distributional surveys of the herpetofauna (reptiles and amphibians) of the Tsaratanana Massif using the same elevational sampling transect and research camps. To the best of our knowledge, these surveys represent the only repeat biological surveys of any montane elevational transect in Madagascar over the past two decades.

The objectives of this study are fivefold: (1) determine if there is evidence for temperature warming trends for northern Madagascar (including the Tsaratanana Massif) based on meteorological observations and gridded monthly mean observations constrained to the period of herpetological surveys; (2) compare these meteorological observations with historical climate simulations; (3) apply a simple elevational range displacement analysis (based on lapse rates) to estimate upslope displacement at Tsaratanana; (4) determine whether there is supporting evidence for current upslope shifts of reptiles and amphibians at Tsaratanana; and (5) assess potential extinction vulnerability from upslope displacement for this and other montane assemblages in Madagascar.

## Materials and methods

### Weather station and reanalysis data

Weather station records in Madagascar are sparse both in space and in time, especially near Tsaratanana and at higher elevations. Therefore, in order to evaluate temperature changes between the 1993 and 2003 herpetological surveys, we used a number of sources, including individual stations, gridded observations, and reanalyses. We considered temperature changes between the following two decades: 1984–1993 and 1994–2003 (i.e. the decade preceding and including the year of each survey). The first decade included three El Niño (warm) and two La Niña (cool) events, while the second period contains three El Niño (warm) and four La Niña (cool) events (Climate Prediction Center, US National Oceanic and Atmospheric Administration, http://www.cpc.ncep.noaa.gov/products/analysis_monitoring/ensostuff/ensoyears.html). In addition, we also checked results with the first period extended to 1982–1993 in order to include the strong warming from the El Niño event of 1982–1983, to confirm this did not remove the temperature trends. Weather station data were annual mean temperature taken from the adjusted Global Historical Climatology Network stations used in Hansen *et al*. (2001). These comprise Diego-Suarez (Antsiranana) (105 m elevation, 190 km from Tsaratanana), Majunga (Mahajanga) (18 m, 340 km), Maintirano (23 m, 690 km), Tulear (Toliara) (8 m, 1180 km), and Antananarivo (Tananarive) (1280 m, 560 km). We also included annual mean minimum and maximum daily temperature observations from Antananarivo obtained directly from the Direction Générale de la Météorologie de Madagascar, Antananarivo. We were unable to locate other stations that were either closer to Tsaratanana or at higher elevations. The gridded data were obtained from the CRUTEM3 (land) and HadCRUT3 (land+ocean) datasets of gridded monthly mean observations on a 5 × 5 degree grid (Brohan *et al*., 2006) (available at: http://www.hadobs.org). We also analysed monthly mean temperature from the ERA-40 (Uppala *et al*., 2005) (available at: http://www.ecmwf.int/research/era/for data through August 2002), NCEP (Kalnay *et al*., 1996), and NCEP2 (Kanamitsu *et al*., 2002) (both available at: http://dss.ucar.edu/datasets/) reanalyses. These are effectively repeats of the historical weather forecast using a standard version of the weather forecast model, with the model interpreting both local station temperature observations and other nonlocal weather observations. For these gridded datasets we considered two regions: northern Madagascar, 46–51°E, 12–17°S; and Madagascar and nearby Indian Ocean, 42–54°E, 9–28°S.

### Historical climate model data

We used temperature output produced by historical simulations of global climate models provided for the CMIP3 archive project (available at: https://esg.llnl.gov:8443/index.jsp) to produce temperature data for the same region and decades as we used for the gridded data. These models simulate the internal natural dynamics of the climate system, such as ENSO, and their response to changes in external forcings. We used 30 historical simulations of nine climate models, selected according to the criteria of Stone *et al*. (2007), and provided that they continued through to the end of 2003. Historical simulations include changes in at least four external forcings: estimated changes in solar luminosity, changing stratospheric aerosols from volcanic eruptions, changing greenhouse gas concentrations, and changing sulphate aerosol concentrations due to anthropogenic activities. For comparison, we also examined data from 33 segments of control simulations of these models in which all external forcings were held constant at ‘preindustrial’ levels.

### Estimating upslope elevation displacement

Temperature trends were used to make predictions of elevational displacement in species distributions using moist adiabatic temperature lapse rates (which describe the relationship between air temperature and elevation). We used two moist adiabatic lapse rates of 5 and 6 °C per 1000 m; these rates have been widely observed and are commonly used by other researchers (e.g. Grabherr *et al*., 1994; Hostetler & Mix, 1999; Walther *et al*., 2002; Wright *et al*., 2004; Rull & Vegas-Vilarrúbia, 2006;). We applied a simple elevational range displacement analysis where species are assumed to track the spatial shifts in the temperature envelopes that they occupy (changes in isotherm height).

### Herpetological surveys at Tsaratanana

The elevational distribution of amphibians and reptile species were recorded during two surveys in 1993 and 2003 on the same transect at Tsaratanana Massif: the trail leading from Mangindrano Village to Maromokotro Summit, Tsaratanana. Sampling was conducted during the Austral summer rainy season (when species activity is expected to be highest) at all elevations between 1400 and 2876 m (the latter representing Maromokotro summit). Previously used field methods were applied, which involved visual searches for animals during the day and at night (with headlamps), in all available habitats below approximately 8 m in canopy height, and the searching of refugia (e.g. plant axils, dead wood, and under rocks, root mat, and leaf litter) (see Raxworthy & Nussbaum, 1994a).

We utilized three main camps to survey this transect (Fig. 1): Befosa (14°10.455′S, 48°56.708′E, 1600 m), Matsabory Maiky (14°09.175′S, 48°57.431′E, 2050 m), and Bepia Ridge-Ambodinitsaratanana (14°04.807′S, 48°59.123′E, 2500 m). Herpetologist team size was 5 in 1993 and 3 or 4 in 2003. The 1993 survey included a total of 60 herpetologist days (HD) (each day including both daytime and night-time surveying) and the 2003 survey included 97 HD of survey. Camp dates, HD, and recorded maximum and minimum temperature extremes are as follows. 1993: Befosa 31 March to 2 April, 15 HD, 11–21 °C; Matsabory Maiky 22–25 March and 30 March, 25 HD, 11–21 °C; Bepia Ridge-Ambodinitsaratanana 26–29 March, 20 HD, 9–18 °C. 2003: Befosa 23 February to 1 March, 21 HD, 14–23 °C; Matsabory Maiky 1–11 February, 44 HD, 10–21 °C; Bepia Ridge-Ambodinitsaratanana 12–21 February, 32 HD, 11–20 °C. The percentage of HD survey effort devoted to each camp for 1993 and 2003, respectively, were: Befosa 25% and 22%, Matsabory Maiky 42% and 45%, and Bepia Ridge-Ambodinitsaratanana 33% and 33%.

Elevation for each capture was measured to the closest 10 m with altimeters calibrated daily to the same set point of the occupied camp or Maromokotro summit (2876 m). Calibration point elevations were determined using the Foiben Taosarintanin'I Madagasikara (FTM) 1 : 100 000 topographic map (sheet U-35) and checked with GPS units. In those cases where an elevational range was recorded for a series of specimens in the field, we used the midpoint elevation for analyses using these observations, unless other field documentation was also provided. Almost all observations are based on collected specimens that are now deposited at the University of Antananarivo Department of Animal Biology (UADBA), American Museum of Natural History and the University of Michigan Museum of Zoology; field tags: RAN (Ronald A. Nussbaum) collected in 1993; RAX (Christopher J. Raxworthy) collected in 2003 (see Appendix A for complete collection list, and additional observations). In addition, we also examined other herpetological specimens at the Muséum National d'Histoire Naturelle, Paris (MNHP) that were collected with associated elevational data from Tsaratanana (see Appendix A).

Elevational shifts between 1993 and 2003 were tested using the Wilcoxon signed-rank test (one-tailed test for positive change) and the Mann–Whitney test. Differences in elevation shifts between amphibians and reptiles were tested using the Mann–Whitney test, and linear relationships tested using Pearson's correlation.

## Results

### Temperature changes between the decades 1984–1993 and 1994–2003

Because the surveys were separated by 10 years, we consider the simple case where the surveyed species respond to the average temperature over the 10 years before and including the survey years. The average temperature differences between 1984–1993 and 1994–2003 for the five cities and the two regional areas of Madagascar are shown in Fig. 3. The spread between the different datasets and the two regions gives an indication of the importance of local variations in the overall warming pattern. However, regardless of regional variation, we find that warming is evident in every historical dataset. The lowest warming is seen for the town of Diego-Suarez and the highest warming for the towns of Antananarivo, Tulear, Maintirano, and the northern regional area of Madagascar. This latter region is estimated to have experienced temperature increases of between 0.10 and 0.37 °C (Fig. 3a). For the larger Madagascar and Indian Ocean grid (42–54°E, 9–28°S), which includes substantial ocean area, the dampening effect of the ocean is evident, with temperature increases that are about half the magnitude of those in northern Madagascar during this time period (Fig. 3b). These changes emerged despite the fact that both periods included three El Niño events (when local temperatures tend to be warm) while the second period included more (four vs. two) La Niña events (when local temperatures tend to be cool; see ‘Materials and methods’). Very similar warming trends are also evident when we included the strong 1982–1983 El Niño event, by extending the first period to 1982–1993 (see Appendix B); in this case, for northern Madagascar, temperature increases ranged from 0 °C (Diego-Suarez town) to 0.35 °C for the regional area. These observed and estimated changes are also consistent with results for the historical simulations of the CMIP3 climate models, which generally show an increase in temperature between these decades. On the other hand, they are in some cases inconsistent with results from the control simulations and their approximately zero trend. It should be noted that these climate model ranges might be underestimated (see ‘Discussion’).

### Predicted upslope distribution shifts

Applying a simple elevational range displacement analysis to these temperature increases predicts the following upslope displacement. Using lapse rates of 6 and 5 °C per 1000 m, and the northern Madagascar gridded temperature increases (from 0.10 to 0.37 °C) between 1984–1993 and 1994–2003, results in predicted upslope distribution shifts of 17–62 and 20–74 m, respectively, for northern Madagascar.

### Species elevational distributions at Tsaratanana between 1993 and 2003

A total of 30 species (11 reptiles, 19 amphibians), recorded above 1400 m from both the 1993 and 2003 surveys, were considered sufficiently common that their elevational distributions could be compared between these surveys (they were represented by at least four observations, which we applied here as a minimum arbitrary threshold). The elevational distributions of these species are shown in Table 1 (see also Appendix A). An additional 20 herpetological species were also recorded above 1400 m on this transect, but observations for these species were considered insufficient (<4) for making comparisons between the surveys. These included species primarily distributed below 1400 m elevation, secretive and difficult to sample species (fossorial or arboreal), species that occur naturally at low densities (e.g. snakes), and several high-montane species that may now be very limited in distribution (see ‘Discussion’). For the 1993 collection some specimen identifications have been subsequently revised since Raxworthy & Nussbaum (1994b). None of the 30 resampled species were recorded in lower elevation forest (1170–1400 m) along this transect, and forest below 1170 m elevation no longer exists in this region.

**Table 1:** The elevational distribution of 30 species of amphibians and reptiles from Tsaratanana Mountain (1400–2876 m), sampled in 1993 and 2003

		Elevation distribution (m)				Elevation change (m)				
		1993		2003		1993–2003				
Species	Observations	Min.	Max.	Min.	Max.	Min.	Max.	Mid.	Mean.	Med.
Amphibia										
Microhylidae										
*Plethodontohyla guntherpetersi*	7	1450	2700	2300	2500	850	−200	325	–	–
*Plethodontohyla* sp. P	6	2050	2050	2050	2300	0	250	125	–	–
*Platypelis alticola*	7	2350	2350	2300	2600	−50	250	100	–	–
*Platypelis pollicaris*	14	1650	2350	2350	2450	700	100	400	410*	325
*Platypelis* sp. F	7	1420	1650	1580	1680	160	30	95	–	–
*Platypelis* sp. O	6	2050	2350	2000	2550	−50	200	25	–	–
Mantellidae										
*Aglyptodactylus madagascariensis*	31	1450	2075	2000	2050	550	−25	262	111*	−25
*Boophis anjanaharibensis*	11	1625	2050	1610	1950	−15	−100	−58	–	–
*Boophis marojeziensis*	25	1600	1625	1600	1680	0	55	27	–	–
*Boophis* cf. *burgeri*	30	1600	1650	1580	1680	−20	30	5	−17*	−25
*Boophis* sp. A	4	2050	2050	2050	2050	0	0	0	–	–
*Blommersia* cf. *wittei*	5	1630	2100	1900	2050	270	−50	110	–	–
*Gephyromantis asper*	20	1450	2050	1580	2050	130	0	65	171	300
*Gephyromantis tandroka*	16	1625	2000	1620	1670	−5	−330	−168	–	–
*Guibemantis liber*	14	1450	1600	1620	2050	170	450	310	–	–
*Guibemantis* sp. A	11	2050	2350	2050	2050	0	−300	−150	–	–
*Mantidactylus femoralis*	43	1625	2650	1580	2725	−45	75	15	43*	60
*Mantidactylus* cf. *opiparis*	8	1630	1630	1580	1680	−50	50	0	–	–
*Spinomantis* cf. *peraccae*	43	1615	2050	1610	2500	−5	450	222	197*	325
Reptilia										
Chamaeleonidae										
*Brookesia lolontany*	18	1600†	2050	1600	2100	0	50	25	–	–
*Calumma guibe*	22	2050	2100	1975	2250	−75	150	38	–	–
*Calumma* cf. *guillaumeti*	10	1600	1630	1650	1700	50	70	60	–	–
*Calumma* cf. *linota*	14	1600	1650	1635	1675	35	25	30	–	–
*Calumma malthe*	28	1500	1630	1580	1650	80	20	50	25*	25
*Calummapeltierorum*	57	2050	2350	1975	2580	−75	230	78	9	50
*Calumma tsaratananensis*	41	2750	2750	2500	2850	−250	100	−75	–	–
Gekkonidae										
*Phelsuma lineata punctulata*	35	2700	2850†	2600	2850	−100	0	−50	−16*	0
*Uroplatus* sp. A	11	2050	2050	1950	2325	−100	275	88	–	–
Scincidae										
*Amphiglossus melanopleura*	5	1450	1650	1580	1680	130	30	80	–	–
*Amphiglossus tsaratananensis*	7	2050	2700	2050	2550	0	−150	−75	–	–

Changes in species mean and median elevations listed when ≥7 observations for both 1993 and 2003.

min., minimum; max., maximum; mid., mid-point; med., median.

* *P*<0.05.

† See Appendix A.

Between 1993 and 2003, of the 30 resampled species, 17 species showed an increase in overall elevational range, one species showed no change, and 12 species showed a decrease in their overall elevation range (Table 1). The mean for both the minimum and maximum elevations increased between 1993 and 2003. For minimum elevation there was a mean increase of 76.2 m (median=0 m, *n*=30 species, Wilcoxon's signed-rank test: 11 increases, six ties, 13 decreases, *Z*=−1.044, *P*=0.149). There were slightly more decreases than increases for minimum elevation, but most decreases were small in comparison with the increases: eight of 13 decreases ≤50 m, compared with two of 11 increases ≤50 m, with *Plethodontohyla guntherpetersi*, *Platypelis pollicaris*, and *Aglyptodactylus madagascariensis* showing the largest increases (≥550 m). For maximum elevation, there was a mean increase of 57.8 m (median=40.0 m, *n*=30 species, Wilcoxon's signed-rank test: 20 increases, three ties, seven decreases, *Z*=−1.935, *P*=0.027).

These results show an overall trend for asymmetrical rates of invasion and contraction for both upslope and downslope movements, with 20 species exhibiting upslope shifts at the upper margin (range expansion) compared with 11 species exhibiting upslope shifts at the lower margin (range contraction). For downslope shifts, 13 species show a decrease in minimum elevation (range expansion) and seven species show a decrease in maximum elevation (range contraction). Our results find seven of 30 species showing upslope displacement, with both their minimum and maximum elevations moving upwards. By contrast, we find only two species showing downslope displacement with both minimum and maximum elevations decreasing.

**Fig. 4:**
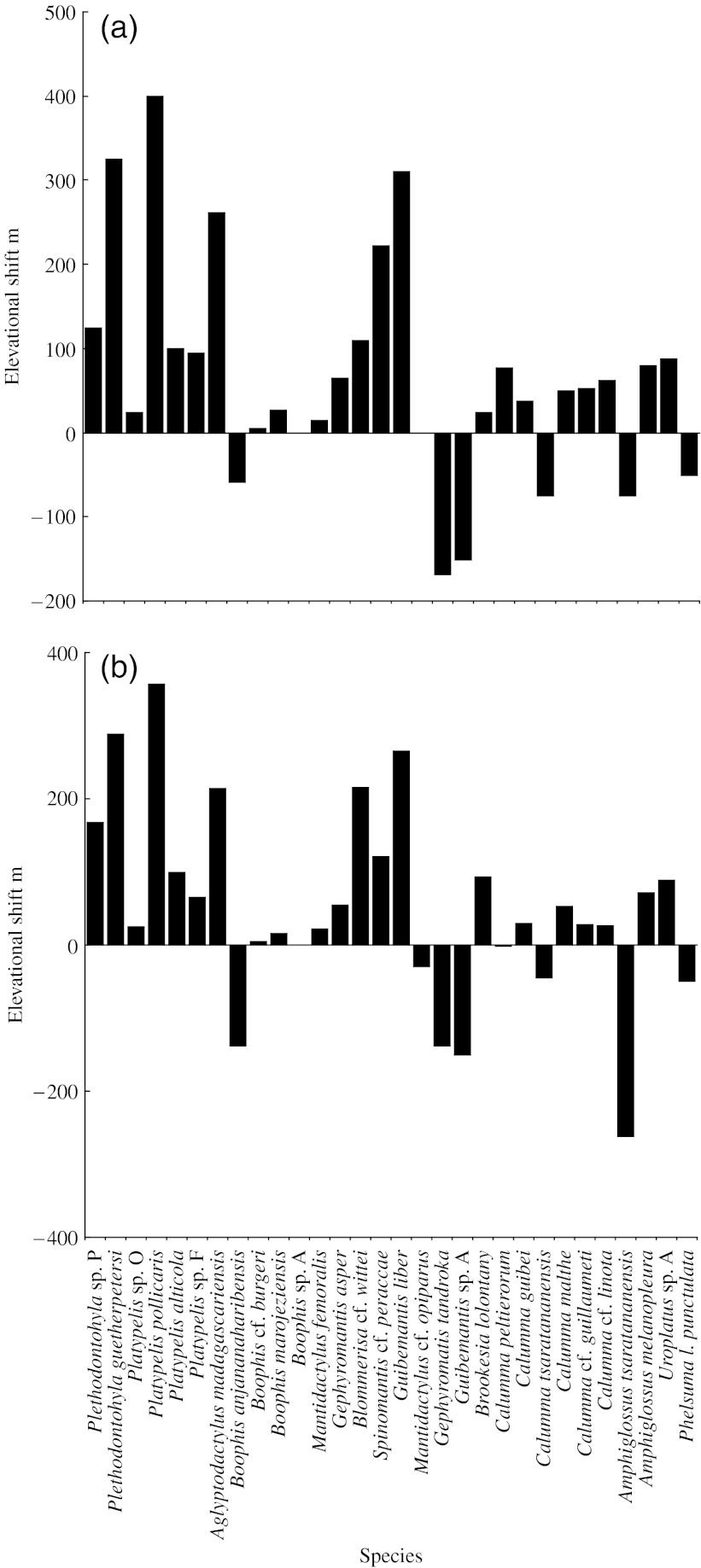
Change in the species midpoint elevation between 1993 and 2003 for 30 species of reptiles and amphibians surveyed at Tsaratanana; (a) all records from both surveys; (b) elevation equalized records for both surveys (mean shifts of five random data samples, see ‘Materials and methods’).

The midpoint elevation (midpoint between minimum and maximum elevation) significantly increased for these species between 1993 and 2003, with a mean elevational increase of 65.3 m (median=44.0 m, *n*=30 species, Wilcoxon's signed-rank test: 22 increases, two ties, six decreases, *Z*=−2.562, *P*=0.005) (Fig. 4a). Comparing the upslope distribution shifts between amphibians and reptiles, this shift was greater in amphibians (amphibian mean change=90 m, median=65.0 m; reptiles mean change=25.0 m, median=50.0 m), but this difference was not significant (Mann–Whitney test, *U*=80.500, *Z*=−1.033, *P*=0.302).

To further explore potential elevation shifts, we also compared the mean and median elevations of observations between 1993 and 2003 for each of the best-sampled species which had ≥7 observations for both years (nine species). An upslope change in mean and median elevation was found in seven and six of these nine species (see Table 1), with an overall mean upslope shift of means and medians of 104 and 115 m, respectively. Differences in the observational elevations between years were significant for seven of the nine species (Mann–Whitney test): *P. pollicaris*, *U*=1.000, *Z*=−3.071, *P*=0.001; *A. madagascariensis*, *U*=73.500, *Z*=−1.884, *P*=0.030; *Boophis* cf. *burgeri*, *U*=24.000, *Z*=−3.949, *P*=0.000; *Mantidactylus femoralis*, *U*=63.000, *Z*=−2.052, *P*=0.020; *Spinomantis* cf. *peraccae*, *U*=102.500, *Z*=−2.080, *P*=0.019; *Calumma malthe*, *U*=43.000, *Z*=−2.281, *P*=0.012; *Phelsuma l. punctulata*, *U*=104.000, *Z*=−2.434, *P*=0.008.

**Fig. 5:**
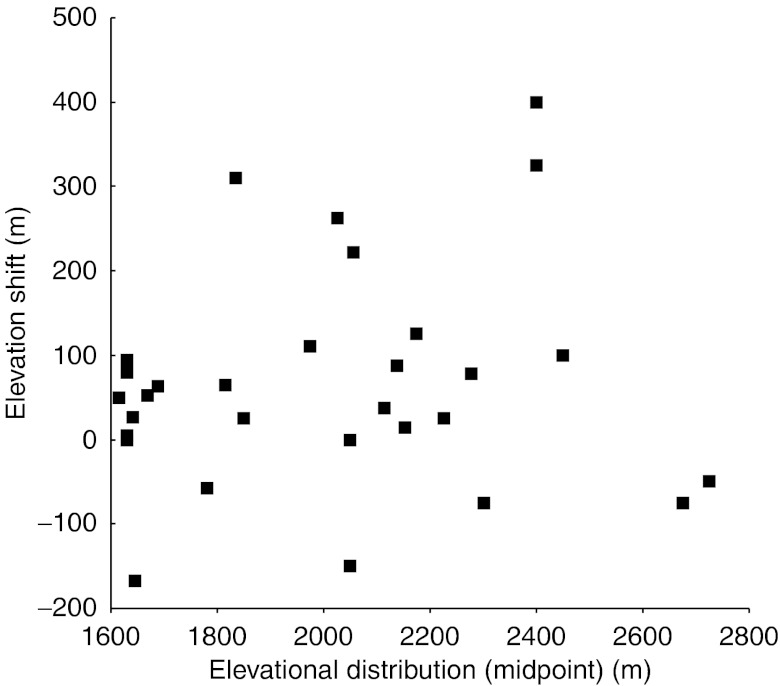
Elevation shifts in the distribution midpoint between 1993 and 2003 plotted against the 1993 midpoint elevation for 30 species of reptiles and amphibians.

Although the same research camps were used in both surveys, with sampling inevitably clumped around the elevational range of each camp, there was also the potential for elevationally biased resampling. To evaluate possible elevational bias, we conducted the following evaluations. First, we examined the distribution of species elevational shifts by elevation. We did this because elevationally biased resampling could result in a biased elevational distribution for range shifts (e.g. if sampling was biased towards higher elevations, then shifts would be larger at higher elevations). We found that species exhibiting upslope shifts are distributed across almost the entire elevational transect, and no obvious elevational trend is evident (Fig. 5). There was no significant relationship between species elevational distribution (1993 midpoint) and elevational shift (mid, minimum, or maximum) in any of the analyses (*n*=30; midpoint elevation: *r*=0.127, *P*=0.503; minimum elevation: *r*=0.040, *P*=0.835; maximum elevation: *r*=0.146, *P*=0.441).

Second, we conducted a reduced data analysis where we equalized elevational records between surveys for each 200 m elevation band (see also Warren *et al*., 2001, for a similar method of equalized recorder effort). We capped the number of records (for the 30 resampled species) to the minimum number collected in each elevation band by either survey. Discarded records (mostly from the 2003 survey) were randomly selected from within each elevational band, and we repeated this random sampling procedure five times. As a result of these data reductions, a total of 169 records from each survey were included in each of the five reduced datasets. As previously, we also only compared species with at least four observations, resulting in 23–28 species being included in these repeat analyses. For the minimum species elevation, in all five analyses there was positive mean upslope shift of between 28.8 and 114.0 m (mean=59.2 m, median=54.6 m); Wilcoxon's signed-rank tests, six to 12 increases, five to seven ties, nine to 12 decreases, *Z*=0 to −2.035, *P*=0.021–0.500, with one run with *P*<0.05. For the maximum species elevation, in four of five analyses there was a positive mean upslope shift between −8.4 and 53.5 m (mean=19.4 m, median=8.5 m); Wilcoxon's signed-rank tests, 14–17 increases, three to four ties, four to 10 decreases, *Z*=−0.014 to 1.874, *P*=0.031–0.495, with one run with *P*<0.05. For the midpoint species elevation, in all five analyses, there was positive mean upslope shift of between 18.8 and 50.8 m (mean=36.6 m, median=38.8 m); Wilcoxon's signed-rank tests 15–19 increases, one to two ties, five to nine decreases, *Z*=−1.014 to 2.127, *P*=0.017–0.155, with two runs with *P*<0.05. The mean shifts for each species (based on the five analyses) are shown in Fig. 4b. Positive upslope species shifts remained prevalent in these analyses, but the magnitude was decreased, and statistical significance for these elevation-equalized data subsets (with fewer test pairs) was lower compared with the complete dataset.

We also used the equalized elevational records to compare the mean elevations between 1993 and 2003 for the best-sampled species with ≥7 observations for both years in all five data subsets. Three species had sufficient samples sizes, and there was a corresponding upslope change in mean elevation between 1993 and 2003 in 13 of the 15 analyses (Mann–Whitney test): *Spinomantis* cf. *peraccae* mean=42.4 m (*U*=37.5–52.5, *Z*=−0.182 to 1.270, *P*=0.102–0.428); *Calumma peltierorum* mean=42.3 m (*U*=31.5–52.5, *Z*=−0.439 to 1.172, *P*=0.121–0.330), and *C. malthe* mean=19.6 m (*U*=34.0–38.5, *Z*=−0.929 to 1.888, *P*=0.030–0.177).

### Phenology for 1993 and 2003 in northern Madagascar

**Fig. 6:**
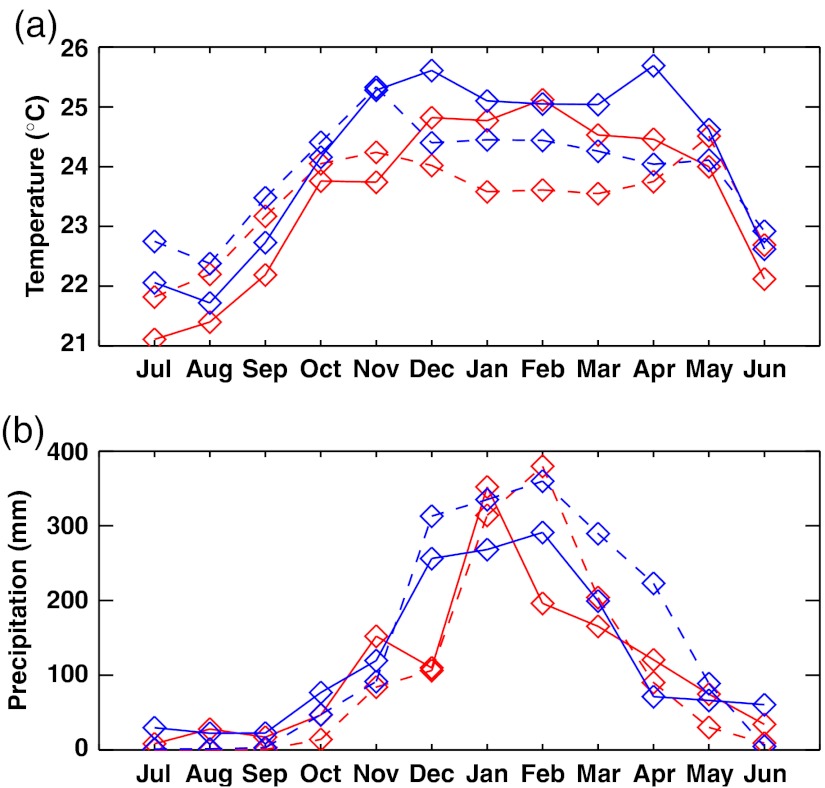
Time series of (a) monthly mean temperature and (b) precipitation over the periods surrounding the two surveys of Tsaratanana. Red lines, 1992–1993 period; blue lines, 2002–2003 period. Data from the NCEP (solid) and NCEP2 (dashed) reanalyses for the grid area 47.8–49.7°E and 15.2–13.3°S.

Mean temperatures during both the survey periods (22 March to 2 April 1993 and 1 February to 1 March 2003), and the preceding month before each survey period, were all very similar (Fig. 6). Almost no difference is evident in the NCEP data, and <1 °C difference evident in the NCEP2 data. These results are also in agreement with the field temperature data that we recorded at each survey camp for 1993 and 2003 (see ‘Materials and methods’). For monthly precipitation, the March rainfall in northern Madagascar was similar in both 1993 and 2003, but there may have been a larger difference for February between these years. Concerning our survey dates, this grid region had 165–204 mm precipitation in March 1993, and 291–360 mm in February 2003. For both datasets, this amounts to March 1993 receiving 57% as much rain as in February 2003. However, rainfall was much more similar in January for both years, with this month having either the highest or second highest monthly rainfall in both these rainy seasons.

In summary, therefore, there was little difference in the monthly temperatures between the 1993 and 2003 surveys, and for both years, the onset of the rainy season was already well underway by the month of January (the preceding month or 2 months before both surveys). Both surveys fall within the wettest quarter of each year (during the peak rainy seasons), but we do find evidence for a difference in rainfall during the actual survey periods.

## Discussion

### Temperature trends and estimated upslope displacement in Northern Madagascar

The warming trends that we report here for Madagascar equal or exceed global averages (Karoly & Wu, 2005; Rosenzweig *et al*., 2007; Trenberth *et al*., 2007;). For northern Madagascar, the gridded observational data show this region experiencing warming between 1993 and 2003 of between 0.10 and 0.37 °C, which is also similar in magnitude to the warming reported for several other weather stations outside this area (Maintirano, Tulear, and Antananarivo). Ideally, we would also have data for the higher elevation areas of the Tsaratanana Massif (or other massifs in Madagascar), but these data do not yet exist (see ‘Materials and methods’). However, we have included data for the highest elevation weather station in Madagascar: Antananarivo, at 1280 m, which is 120 m lower in elevation than the start of the Tsaratanana transect. Warming is evident between these two periods, despite the second period including four vs. two La Niña cooling events (both periods include three El Niño warming events). These warming trends also remained evident when we included the strong 1982–1983 El Niño warming event in the first period (Appendix B). We thus find that these ENSO events do not heavily influence these warming trends over the 10–12-year time period.

These regional warming trends are also consistent with the CMIP3 climate model historical simulations for the same area. Of particular significance, the gridded observational warming trends for Madagascar since the early 1980s are consistent with historical simulations based on changing external forcings, but are sometimes larger than the range of the trends of the control simulations with constant external forcings. It should be noted that the variability of climate models is typically damped at spatial scales near to their grid resolution (such as the regions we examine here) in order to maintain numerical stability, so the ranges we show here are probably underestimated. Nevertheless, these results suggest that the external forcings are required to reproduce this warming trend. Estimates of changes in sulphate aerosol concentrations over this region are small, while solar irradiance changed little over this time (Forster *et al*., 2007). The stratospheric aerosols from the eruptions of El Chichón in 1983 and Pinatubo in 1991 could induce an overall warming trend for the 1984–2003 period we examine here, by cooling the 1984–1993 decade, but none of the simulations show discernible cooling responses over Madagascar to these events (not shown). This leaves only one remaining external driver: emissions of greenhouse gases, which have been increasing steadily over this period.

These findings are consistent with the conclusions of Stott (2003), who detected the effects of anthropogenic forcings in the historical temperature changes over the entire African continent but was unable to detect the effect of natural forcings. One caveat is that most of the historical model simulations do not include the effects of changing land cover and use, which can dramatically alter the albedo and evapotranspirative properties of the surface at local scales. Because land cover is continuing to change over Madagascar as a result of human activity (e.g. Green & Sussman, 1990; Elmqvist *et al*., 2007;), this could potentially contribute to local climate change. Elsewhere, for example, cloud forests in Costa Rica appear to be receiving less moisture due to lowland deforestation producing drier air, which elevates the base height of orographic cloudbanks during the dry season (Nair *et al*., 2003).

The degree of warming we report here for Madagascar can be expected to impact the island's biodiversity in multiple ways, but in particular, this has the potential to drive tropical montane species to higher elevations. Applying the two lapse rates (5–6 °C per 1000 m) and using the gridded observational datasets warming estimates, we estimate an upslope displacement of between 17 and 74 m between 1993 and 2003 in northern Madagascar. By comparison, our survey results for the elevational distribution (midpoint) of reptiles and amphibians at Tsaratanana, find upward trends of 19–51 m (mean 37 m) based on the elevation equalized data subsets; and using all data, mean upslope shift of 65 m. The similarity between our observed upslope elevational trends, and the displacements estimated from temperature trends, is thus supportive for these displacements being driven by warming. In addition, these findings are especially novel in that they describe a tropical montane system, for which otherwise, there is a general dearth of comparative empirical data (IPCC, 2007b). For example, our review of the literature (see ‘Introduction’) failed to find other tropical studies that have measured upslope displacements of species distributions based on repeat transect surveys. And more generally, to date there have been relatively few tropical field studies that have investigated climate change impacts on biodiversity. Consequently, there may be a growing misperception that climate change represents a problem for biodiversity predominantly in the temperate and near-artic zones. Yet perversely, the majority of extinctions driven by climate change are likely to occur in tropical areas, which include both high species richness and narrow endemism, particularly in tropical montane systems.

Nevertheless, we also remain cautious about interpreting these results because of the temporal and spatial limitations of our survey at Tsaratanana, the lack of co-located meteorological measurements specific to the Tsaratanana Massif, and the potential phenological differences that might also account for these apparent upslope survey results.

First, we consider these results to be preliminary because they are based on just two sets of temporal observations (Sparks & Tryjanowski, 2005). In particular, the temporal constraints of this study may have partly contributed to the observed variation in species distribution changes that we describe in the results (although variable species displacement responses have been reported for butterflies, Konvicka *et al*., 2003, and vascular plants, Pauli *et al*., 2007). Our results also show fewer species exhibiting upslope expansion for the lower margin (range contraction) compared to the upper margin (range expansion), which conforms to other observed asymmetrical rates of invasion and contraction seen at species margins, where invasion may occur at a faster rate (Parmesan, 1996; Walther *et al*., 2002;). More temporal observations are desirable to confirm these findings, and we plan to make future repeat surveys of this transect between now and 2013. However, because of the extreme scarcity of empirical results for tropical montane systems, we also think it prudent to present these preliminary results now.

Second, our results are based on just a single massif. Ideally, it would be desirable to have results from comparable montane transects elsewhere in Madagascar. Unfortunately (to the best of our knowledge), such additional data are not yet available for any other montane transect. Although there have been several recently undertaken elevational surveys of species distributions in other montane regions of Madagascar, no repeat surveys have yet been conducted (see edited volumes by Goodman, 1996, 1998, 1999, 2000; Gautier & Goodman, 2002; Goodman & Wilmé, 2003). This latter problem is not unique to Madagascar. As noted by Epps *et al*. (2003) and Rull & Vegas-Vilarrúbia (2006), sparse distribution data continue to hamper our ability to detect elevation displacement for many montane regions of the world.

Third, we cannot completely discount the possibility that phenological differences between 1993 and 2003 may have influenced our survey results (most herpetological species aestivate or hibernate during the cooler and drier Austral winter from May to September). However, we do not consider this likely for the following reasons. Both surveys took place during the last month of the three wettest months of each respective rainy season (see Fig. 6). The explosive-breeding amphibians had already reproduced before both surveys (as evidenced, e.g. by *A. madagascariensis* being in postbreeding condition), but other amphibians were found to be vocalizing and/or represented by gravid females. We also noted that the same streams and volcanic lakes were filled with equivalent quantities of water during both surveys, and that the ground cover was completely saturated with water. Thus, while the regional precipitation during the 1993 survey was about 57% of that in 2003, we found similar local field conditions and amphibian reproductive states. Concerning reptiles, during both surveys, adults were in breeding condition as evidenced by males with fully developed hemipenes and some females being gravid. Temperatures were also similar (Fig. 6), which is significant because seasonal herpetological activity is often directly influenced by temperature (see Zug *et al*., 2001). In addition, the broad taxonomic diversity argues for the results being robust to more subtle phenological differences influencing species activity: these 30 species represent two orders and five families (Table 1).

Comparing amphibians with reptiles, the largest upslope shifts occur with the frogs, especially the two microhylid genera *Plethodontohyla* and *Platypelis*, where all six species show a positive trend (Fig. 4, Table 1). The breeding habits of these species are not yet well known, but these genera are not known to be dependent on stream habitats for egg deposition. By contrast, the four stream-breeding *Boophis* frogs show little change in elevation. However, another stream-breeding frog *Spinomantis* cf. *peraccae* does show a substantial upslope shift. Among the reptiles, upslope shifts are prevalent in six of the seven chameleon species (*Brookesia*, *Calumma*), but otherwise, no other taxonomic or ecological patterns are obviously evident from these data.

### Potential upslope extinction vulnerability at Tsaratanana

Upslope displacement represents an important potential extinction threat to narrow endemic species confined to the very highest elevations of massifs (Rull & Vegas-Vilarrúbia, 2006). These authors applied a simple elevational range displacement analysis to plant species in the Guayana Highlands, where they used lapse rates to predict complete habitat loss for endemic species within the vicinity of summits. The correspondence that we find at Tsaratanana between species upslope trends and estimated displacement (based on warming and lapse rates) supports the general assumption of this method that species track their current temperature envelope. It is also evident that temperature gradients greatly influence herpetological species distributions and that niche space includes temperature-related dimensions (e.g. Raxworthy *et al*., 2003, 2007; Graham *et al*., 2004; Pearson *et al*., 2007). However, like many estimates of extinction vulnerability, a simple displacement analysis also represents an inevitable oversimplification, because it does not take into account other factors such as adaptation, competition, disease, demographics, and other habitat changes (Pearson & Dawson, 2003), or other stochastic influences. We thus cautiously apply a displacement analysis approach here, to provide a preliminary appraisal of upslope extinction vulnerability based on warming.

At Tsaratanana, six species (four amphibians, two reptiles) have only ever been sampled at elevations <600 m below the highest summit at 2876 m. Three of these species were sampled in 1993 and 2003: *Platypelis alticola*, *Calumma tsaratananensis*, and *Phelsuma l. punctulata* (Table 1, Fig. 2), two sampled in 1993 only: *Plethodontohyla* sp. novs. Q. (2300 m) and Z (2700 m) (S. Wu, unpublished results), and one collected just once in 1949: *Platypelis tsaratananensis* (2600 m). Additional specimen data for these latter three species are provided in Appendix A. For the three resampled species, two show upslope expansion at their upper distribution limit (100–250 m) and all three show downslope expansion at their lower distribution limit (50–250 m) between 1993 and 2003, suggesting that these species are expanding their distributions. Conversely, however, the three other species may have declined. They were not resampled during 2003, and indeed for *P. tsaratananensis*, this species has not been observed subsequent to its discovery in 1949. While we certainly consider it premature to consider any of these species extinct (they could have been missed by surveys – each is represented by one to six specimens), nevertheless, their very limited known distribution, and projected declining habitat, indicates potential conservation concern.

Utilizing a simple elevational range displacement analysis, with the more conservative 6 °C per 1000 m lapse rate, finds that a temperature increase of 1.7 °C would be sufficient to lead to complete habitat loss for three species: *Plethodontohyla* sp. Z, *P. tsaratananensis*, and *Phelsuma l. punctulata* (all endemic to the top 300 m elevation band). This is lower than the proposed 2 °C ‘dangerous warming’ threshold (ISSC, 2005). Concerning *Phelsuma l. punctulata*, our observational data suggest that this species has actually expanded its distribution downslope. But for the other two species, they have not been seen since 1993 and 1949, respectively. Based on our observed warming and overall upslope trends, and the mid-range global climate model projections (IPCC, 2007a), these potential upslope extinctions are projected to occur within the next 50–100 years (assuming no lag-time in species responses).

We currently do not have direct evidence for habitat upslope displacement or loss occurring at Tsaratanana. However, observations made during three botanical surveys of the massif by Perrier de la Bâthie (1927) are suggestive that the tree line might have recently moved upslope. During the 1912 survey of the summit region, he recorded relict forest up to a maximum elevation of 2400 m. At this time, he also noted evidence of recent burning and forest loss, and a return visit, in 1924, found that even this forest had been burnt. Because these highest elevation forests in Madagascar were of great botanical interest to him, it can be reliably assumed that he made considerable effort to explore the relatively small summit area for forest; and his elevation records also appear accurate (he recorded the Maromokotro summit elevation as 2880 m, compared with 2876 m on current FTM maps). By 1993, we found forest reaching a maximum elevation of 2600 m at Tsaratanana (Raxworthy & Nussbaum, 1994b). These observations suggest a possible upslope movement of 200 m in 80 years (25 m per decade). However, it remains uncertain as to how much of this possible upslope shift might reflect forest recovery from burning, because we do not know how fires (a natural and anthropogenic occurrence in montane areas of Madagascar) have more recently influenced the vegetation of Tsaratanana.

### Potential upslope extinction vulnerability for other massifs in Madagascar

The general warming temperature trends that we find for Madagascar indicate that the other massifs will be experiencing similar biological responses. The distributions of high-montane endemic species mirror the situation found at Tsaratanana, with locally restricted species confined to small areas within 600 m elevation of the highest summits (Table 2, Fig. 1). Consequently, similar degrees of upslope displacement would result in the extinction of locally endemic species from many of the major massifs in Madagascar.

**Table 2:** Examples of species from other massifs in Madagascar, known only from elevations <600 m from the highest summits within their distribution range

Massif	Species	Group	Source
Andohahela	*Spinomantis guibe*	Amphibians	Nussbaum *et al*. (1999)
	*Calumma capuroni*	Reptiles	Nussbaum *et al*. (1999)
Itremo	*Lygodactylus pauliani*	Reptiles	Pasteur (1991)
Ibity	*Lygodactylus arnoulti*	Reptiles	Pasteur (1967)
	*Lygodactylus blanci*	Reptiles	Pasteur (1967)
	*Arundinaria ibityensis*	Bamboos	Dransfield (2003)
Ankaratra	*Mantidactylus pauliani*	Amphibians	Guibé (1974)
	*Lygodactylus mirabilis*	Reptiles	Raxworthy & Nussbaum (1994b)
Marojejy	*Microgale monticola*	Tenrecs	Goodman *et al*. (2003)
	*Calumma peyrierasi*	Reptiles	Raselimanana *et al*. (2000)
	*Calumma jejy*	Reptiles	Raxworthy & Nussbaum (2006)
	*Blechnum longepetiolatum*	Ferns	Rakotondrainibe (2000)
	*Cheilanthes* sp. nov. 1	Ferns	Rakotondrainibe (2000)
	*Cyathea alticola*	Ferns	Rakotondrainibe (2000)
	*Lindsaea* sp. nov. 1	Ferns	Rakotondrainibe (2000)
	*Arundinaria marojejyensis*	Bamboos	Dransfield (2003)
Anjanaharibe-Sud	*Microgale monticola*	Tenrecs	Goodman *et al*. (2003)
Bemanevika	*Microgale jobihely*	Tenrecs	Goodman *et al*. (2006)
Montage d'Ambre	*Pseudoxyrhopus ambreensis*	Reptiles	Raxworthy & Nussbaum (1994a)
	*Calumma amber*	Reptiles	Raxworthy & Nussbaum (2006)

The distribution of these high-montane endemics, being confined to elevations so close to the highest summits, also indicates that in recent evolutionary time, temperatures in Madagascar have probably not been much warmer than current conditions (see also Hannah *et al*., 2000). And of additional concern, because many species are restricted to primary forests (e.g. Jenkins, 1987), the ongoing fragmentation of natural habitats (e.g. Green & Sussman, 1990; Dufiles, 2003;) is also creating barriers that prevent future dispersal upslope (Walther *et al*., 2002). Although plans for substantial expansion of the national protected area network are currently underway (ANGAP, 2001; Randrianandianina *et al*., 2003;), this does not yet include explicit provisions for warming temperatures and corresponding upslope distribution shifts of species. Our results demonstrate the importance of anticipating and mitigating against upslope distribution shifts (see also Hannah *et al*., 2000). Consequently, high-priority needs to be given to conserving primary habitats that exhibit maximum elevational range, thus making forest blocks associated with the major massifs especially important. Biotic connectivity between protected areas will also be improved by favouring the establishment of corridors that include a broad elevational range.

These displacement extinction threats are not unique to Madagascar: warming trends are widespread (Trenberth *et al*., 2007) and vulnerable endemism is a typical feature of many tropical montane assemblages (Williams *et al*., 2003; Ricketts *et al*., 2005; Pounds *et al*., 2006; Rull & Vegas-Vilarrúbia, 2006;). Although few assemblages have yet been studied in terms of elevational displacement, these upslope shifts are likely to now be occurring in many tropical regions (IPCC, 2007b). For example, a 69 m per decade increase in freezing height (0 °C isotherm altitude) has been recorded for tropical South America (Diaz & Graham, 1996). In addition, for tropical amphibians, emerging infectious diseases combined with climate change may yet further increase extinction threats (Lips *et al*., 2005; Pounds *et al*., 2006;).

However, for most tropical massifs, there are even insufficient survey data to document the current species distributions along elevational transects. We, thus, advocate that surveys of montane endemism should be initiated as soon as possible, to provide the comparative data needed for future monitoring. More specifically, these surveys could readily achieve the following objectives: (1) establish transects for repeat surveys, (2) describe current species elevational distributions, (3) assess species extinction vulnerabilities to upslope displacement based on local meteorological data, and (4) archive baseline distribution data for future monitoring. Although more challenging, the collection of detailed habitat and microhabitat data would also be beneficial, to allow the actual mechanisms of distribution shifts to be better explored. All these efforts will be especially important for massifs with documented or suspected endemism confined to the summit regions, which (extrapolating from the results of our study) may be vulnerable to upslope extinction within a period as short as 50–100 years. Concerning Madagascar, the more recent biodiversity inventories undertaken in other montane regions offer the potential of providing valuable data for future comparisons, assuming that subsequent surveys are completed.

## Figures and Tables

**Fig. B1:**
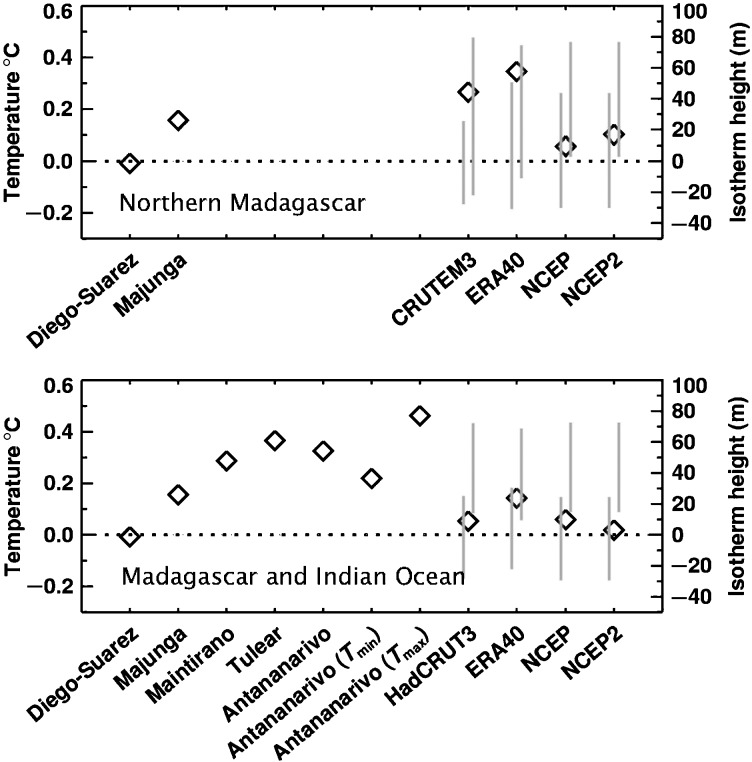
Changes in mean annual temperature between 1982–1993 and 1994–2003 for five major towns in Madagascar and the gridded datasets. Right axis shows the corresponding change in the isotherm height assuming a lapse rate of 6°C per 1000 m. (a) Northern Madagascar: two northern towns (Diego-Suarez and Majunga) and the northern Madagascar regional land grid 46–51°E, 12–17°S; (b) Madagascar and the Malagasy Indian Ocean region: five towns distributed throughout Madagascar and the Madagascar regional land+ocean grid 42–54°E, 9–28°S. Values from observational datasets are plotted as diamonds. For each gridded observational dataset, values from model simulations (see ‘Materials and methods’) are plotted as lines denoting the approximate 5th–95th percentile range with control/historical simulations used for the left/right lines, respectively. Model data are retained for the calculation only where and when the respective observational dataset has data.

## Appendix A: Specimens and observations

### Amphibia

*Plethodontohyla guetherpetersi*: RAN 43244, 43259, 43278, 43294, 43391; RAX 5616, 5687. *Plethodontohyla* sp. P: RAN 43219, 43239; RAX 5385, 5746, 5747, 5748. *Plethodontohyla* sp. Q: RAN 43249 (2300 m elevation). *Plethodontohyla* sp. Z: RAN 43277 (2700 m elevation). *Platypelis tsaratananensis*: MNHP A685-690 (collected by Paulian in 1949 at 2600 m elevation; Guibé, 1974). *Platypelis alticola*: RAN 43250, 43251; RAX 5615, 5626, 5672, 5709, 5741. *Platypelis pollicaris*: RAN 43221, 43225, 43226, 43227, 43252, 43363, 43364; RAX 5613, 5614, 5740, 5742, 5743, 5744, 5745. *Platypelis* sp. F: RAN 43353, 43361, 43400; RAX 5792, 5878, 5880-81. *Platypelis* sp. O: RAN 43222, 43253, 43254; RAX 5592, 5660, 5736. *Aglyptodactylus madagascariensis*: RAN 43200, 43207-08, 43211-16, 43290, 43392-94; RAX 5311, 5397, 5398, 5420, 5471, 5486, 5487-88, 5489, 5496, 5541-42, 5543, 5549-50, 5569-70, 5571. *Boophis anjanaharibensis*: RAN 43223, 43345-47, 43367-68; RAX 5417-18, 5789, 5954, 5955. *Boophis marojeziensis*: RAN 43348-52, 43369; RAX 5754, 5757, 5758, 5759, 5760, 5761-63, 5832, 5833, 5950-52, 5957-59, 5960, 5961, 5962. *Boophis* cf. *burgeri*: RAN 43338-44, 43362, 43370-76; RAX 5765-66, 5767, 5768, 5825-26, 5827, 5828, 5829, 5830, 5910. *Boophis* sp. A: RAN 43199, 43210, RAX 5577, 5595. *Blommerisa* cf. *wittei*: RAN 43218, 43312; RAX 5325, 5580, 5602. *Mantidactylus* cf. *opiparis*: RAN 43309, 43310-11; RAX 5756, 5764, 5845, 5930, 5931. *Gephyromantis asper*: RAN 43224, 43299, 43307, 43355, 43357, 43379, 43395; RAX 5314, 5322,5323, 5324, 5419-20, 5470, 5491, 5508, 5604, 5605, 5885, 5979. *Gephyromantis* cf. *tandroka*: RAN 43306, 43308, 43354, 43380; RAX 5820-21, 5839, 5840-41, 5912, 5913, 5945, 5946, 5947-48, 5970. *Guibemantis liber*: RAN 43359-60, 43396-98; RAX 5495, 5574, 5791, 5877, 5882, 5883-84, 5932, 5978. *Guibemantis* sp. A: RAN 43217, 43233-35, 43248; RAX 5312-13, 5469, 5548, 5575-76. *Mantidactylus femoralis*: RAN 43276, 43303-05, 43356, 43365-66; RAX 5310, 5400, 5401-02, 5403, 5404, 5597, 5598-601, 5661-62, 5750-53, 5769,5770-71, 5772-73, 5774-76, 5777, 5778-82, 5842-43, 5844, 5949. *Spinomantis* cf. *peraccae*: RAN 43209, 43242, 43313-19, 43377-78; RAX 5396, 5405, 5406, 5407, 5408, 5409-12,5413-14, 5415-16, 5490, 5596, 5642, 5643, 5734, 5735, 5755, 5783, 5784-85, 5786, 5787-88, 5835, 5836-37, 5838, 5911, 5963.

### Reptilia

*Brookesia lolontany*: RAN 43292, 43381 (reported in error as 1650 m by Raxworthy & Nussbaum, 1994b; correctly reported by Raxworthy & Nussbaum, 1995); RAX 5393-94, 5472, 5473, 5474, 5482-83, 5484, 5497, 5503, 5504, 5514, 5551, 5572, 5793, 5977. *Calumma guibe*: RAN 43230-1, 43243, 43291; RAX 5387-90, 5479, 5480-81, 5498, 5505, 5506, 5509, 5510-13, 5560, 5561, 5593. *Calumma tsaratananensis*: RAN 43279-81; RAX 5628-29, 5630-32, 5633, 5634, 5635, 5636, 5637-41, 5663-65, 5666, 5667-70, 5676, 5677, 5689-90, 5691-92, 5704, 5705, 5706, 5707, 5708, 5710, 5721, 5722-23. *Calumma malthe*: RAN 43328-34, 43386, 43401; RAX 5846, 5847, 5848-51, 5871, 5915, 5916, 5917-18, 5919, 5920, 5921, 5922, 5923-25, 5974. *Calumma* cf. *guillaumeti*: RAN 43320-24, 43384-5, RAX 5873, 5874, 5926. *Calumma* cf. *linota*: RAN 43326-27, 43390; RAX 5875, 5876, 5927-29, 5968-69. *Calumma peltierorum*: RAN 432001-6, 43255-57; RAX 5326-28, 5329-33, 5334-36, 5337, 5338-40, 5462, 5463, 5464-68, 5475-76, 5477, 5492-94 5498-502, 5515, 5552, 5553, 5554, 5556-59, 5617, 5618-19, 5622, 5623-24, 5625, 5674-75, 5724. *Phelsuma l. punctulata*: RAN 43261-73, 43282-87 (reported in error as 2870 m by Raxworthy & Nussbaum, 1994b); RAX 5644, 5645-46, 5647, 5648, 5649, 5650-51, 5652, 5653, 5654-56, 5657-58, 5659. *Uroplatus* sp. A: RAN 43228-29; RAX 5391, 5392, 5461, 5485, 5507, 5539, 5566-67, 5568. *Amphiglossus melanopleura*: RAN 43197, 43389, 43399; RAX 5909, 5879. *Amphiglossus tsaratananensis*: obs.15.2.03 at 2550 m, RAN 43240-41, 43274, 43289; RAX 5386, 5460.
